# Comparative Time Series RNA-seq Analysis of Pigeonpea Root Tissues in Response to *Fusarium udum* Infection

**DOI:** 10.3389/ffunb.2021.664953

**Published:** 2021-05-17

**Authors:** Khela Ram Soren, Sandhya Tripathi, Shalini Pareek, Maloti Hembram, Priyanka Gangwar, Shrutika Abrol, Abhishek Bohra, Kuldeep Kumar, Aravind K. Konda, P. S. Shanmugavadivel, Md. Akram, Raj K. Mishra, Narendra Pratap Singh

**Affiliations:** ^1^Indian Council of Agricultural Research-Indian Institute of Pulses Research, Kanpur, India; ^2^Department of Botany, Delhi University, New Delhi, India; ^3^Department of Biotechnology, Amity University, Lucknow, India

**Keywords:** *Cajanus cajan*, *Fusarium wilt*, transcriptome, differential gene expression, fungal biotic stress

## Introduction

Pigeonpea [*Cajanus cajan* (L.) Millsp.] is an important food legume and is mostly cultivated in tropical and subtropical regions of South Asia, Kenya, Malawi, Bangladesh, and other parts of the world. India is the center of origin and major global producer (66%), consumer, and importer, ahead of production in Africa (14%). *Fusarium udum* is the causal agent of *Fusarium* wilt of pigeonpea, resulting in severe losses in yield and quality of the produce. The disease is soil-borne and has been reported throughout the world. This crop plays a pivotal role in global food security, balanced diets, and subsistence agriculture because of its diverse uses in food, fodder, fuel, soil conservation, integrated farming systems, and symbiotic nitrogen fixation. The Pigeonpea crop lasts 145–150 days in the field, exposing it to a number of soil-borne and foliar diseases such as wilt, Sterility Mosaic Disease, and Phytophthora leaf spot. Fusarium wilt (FW) is a vascular disease that is common in India's north and central regions, causing substantial yield losses ranging from 30 to 100 percent (Reddy et al., 1990; Hillocks et al., 2010; Kumar et al., 2020). With current crop rotation and fungicide spraying practices, management of FW disease is challenging. Furthermore, these alternatives may have little success because of rapid re-colonization of *F. udum*. In addition, recommended fungicides are costly and spraying results in detrimental effects on the environment, eventually leading to the emergence of new variants of resistant strains of pathogens, thereby, increasing the regulatory concerns against the use of such chemicals. Few FW resistance sources exist among different pigeonpea species, and the majority is inherited quantitatively. Understanding the gene networks and metabolite regulators under action during pathogenesis is essential to facilitate the exploration of plant-fungus interaction pathways and underpin the associated genes/metabolic pathway.

In various plant-pathogen interactions, host susceptibility and tolerance properties are the major determinants of disease progression through spread and growth in plant tissues (Soren et al., 2016). Pathogens have evolved several strategies to affect the host, and plants have evolved numerous physical and chemical barriers, resulting in a sophisticated two-tiered immune system, also known as the zig-zag mode of resistance. The first tier is based on the sensitive perception of pathogen-associated molecular patterns (PAMPs) through pattern recognition receptors (PRRs) on the cell surface of the plant and development of pathogen triggered immunity (PTI). Successful pathogens have evolved to produce effectors to inhibit PTI, and plants may perceive such effectors through additional receptors (R proteins) to form the second tier of defense, effector-triggered immunity (ETI). Undoubtedly, plants and pathogens co-evolve in a complex way, and this dynamic process is continuing, as some pathogens have developed effectors that interact with ETI (Jones and Dangl, 2006).

The plant responds to these pathogens in both qualitative (R genes) and quantitative (multiple genes) ways, with each gene acting in an additive manner. Gene-mediated resistance also activates salicylic acid (SA) dependent signaling pathway which further leads to activation of the effector genes throughout the plant to develop systemic acquired resistance (SAR) against pathogen infection. However, these mechanisms are still poorly understood at the molecular level, despite the fact that they may provide newer insights into the host-pathogen relationship and serve as a guide for potential breeding programs aimed at developing stable and resistant varieties. Furthermore, global gene expression profiling has been successfully demonstrated in a few pulse crops, such as chickpea for herbicide resistance (Iquebal et al., 2017), pigeonpea (Kudapa et al., 2012), and other medicinal crops (Tripathi et al., 2016, 2020). In the present study, we used high-throughput RNA-seq sequencing to perform a comprehensive study of the major differentially expressed genes that were up- or down regulated to better understand the mechanisms and the underlying metabolic pathways impacted by wilt disease of pigeonpea. Two contrasting genotypes of Pigeonpea, Bahar (wilt susceptible) and KPL-44 (wilt resistant), were exposed to the pathogen *F. udum* at different time intervals after infection (0, 72, and 96 h) to identify potential regulatory genes involved in complex spatial-temporal genome regulation for fungal disease resistance.

## Materials and Methods

### Standardization of Pathogen Infection Time Point

The pathogen (*F. udum*) was artificially inoculated onto 30-day-old pigeonpea seedlings (T; 25°C ± 2°C, RH; 60–70%; Photoperiod 14 h and light intensity 500 Lux) to study the histology of host-pathogen interaction. A single spore-derived culture of the virulent *Fusarium udum* (FU12) strain was inoculated in broth culture and shaken in an incubator–shaker at 25°C/120 rpm for 6–7 days. The conidial suspension of FU12 was diluted with sterile water under aseptic conditions to obtain a final concentration of ~6 × 10^5^spores/ml. The 30 day old pigeonpea seedlings, grown in germination trays containing vermiculite and cocopeat (1:1), were carefully uprooted, thoroughly washed in running water, and then washed again with autoclaved water before being dipped in the diluted suspension of fungal inoculums for 5 min to enable the pathogens to enter (Pande et al., 2012). The microtome study of roots was conducted at different time intervals of infected live tissues and visualized under the compound microscope. The minimum threshold time of 24 h after sporulation was established for primary association of spores with the root hairs. Furthermore, after 72 h, sporogenesis begins, and mycelium penetrates the cortical cell, gradually spreading to the entire xylem tissue and the dynamics of transcriptome in the root tissues under this time regime was studied using RNA-seq analysis.

### Next Generation Sequencing (NGS)

Root tissues (2.5 cm long) were harvested at 0, 72, and 96 h after inoculation onto susceptible and resistant genotypes and frozen immediately in liquid nitrogen before being stored at −80°C. RNA was isolated from frozen root tissue samples as per manual instructions provided with RNeasy ®Plant Mini kit (Qiagen). Samples with RIN value > 8 were used for library preparation using Illumina TruSeq RNA Sample prep KitV2 as described in the manufacturers protocol. Libraries were loaded into the Illumina platform (Illumina Highseq2500) for cluster generation and 2 × 150 bp paired end sequencing after the Qubit concentration for the libraries and the mean peak size from the bioanalyser profile were obtained. Paired-End sequencing allows the template fragments to be sequenced in both forward and reverse directions. The description of the NGS experiment is given in a table, along with QC parameter for reads (Supplementary File 1). Furthermore, the transcriptome reads were mapped onto the publicly available *Cajanus cajan*V_1.0 genome (Varshney et al., 2012) to obtain the desired contigs and annotated accordingly (Bio project: PRJNA168332). CLC Genomics Workbench V.9.0 was used for strand specific mapping, and expression values were thus retrieved in terms of total counts.

### Analysis Performed

**Figure 1:**
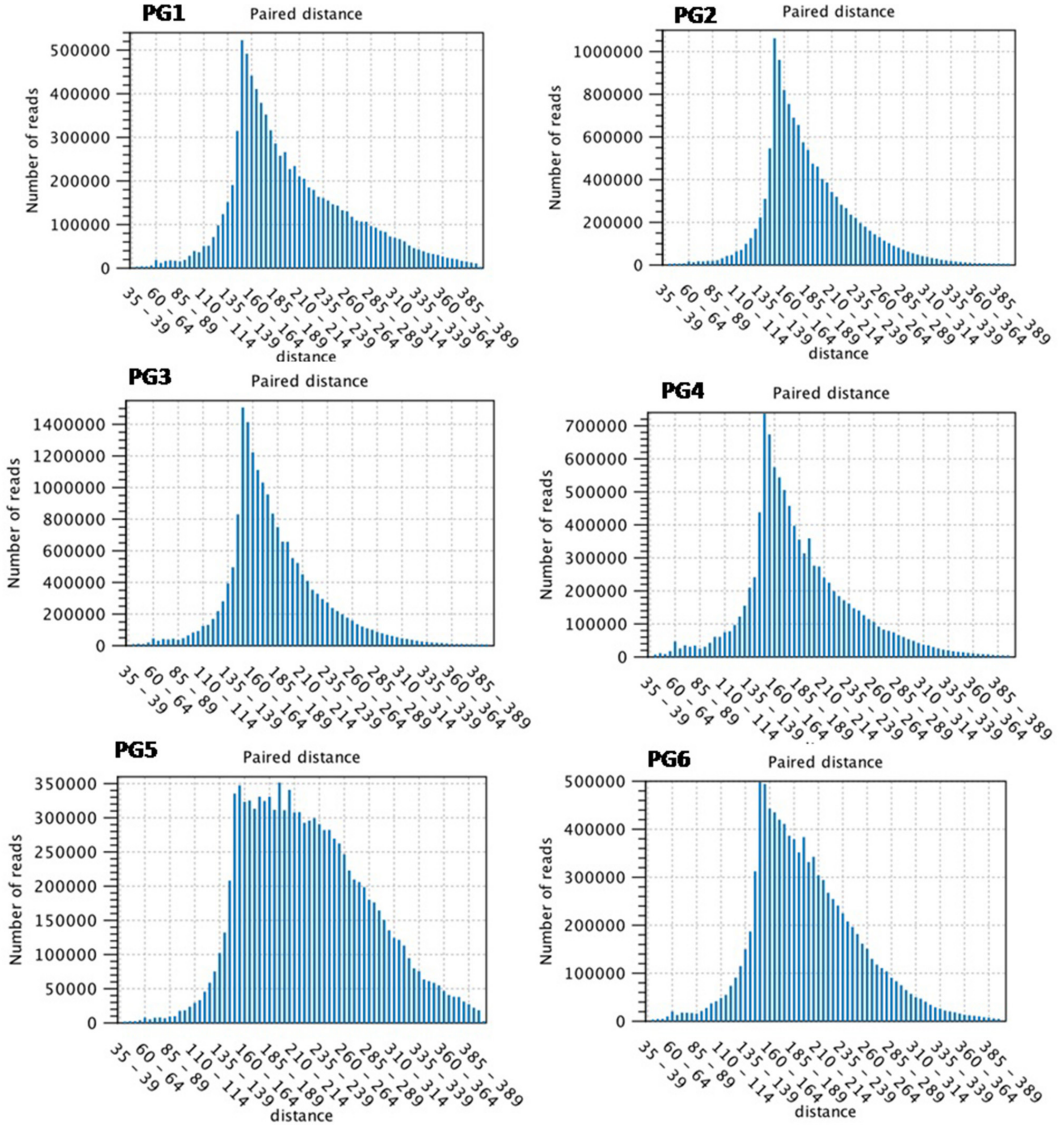
Distribution of paired distance in sequence reads amongst various transcriptome (PG1–PG6) generated through RNA-seq analysis.

The quality control (QC) for raw sequenced reads proceeded with stringent quality control parameters. Removal of adapter, poly-Ns, and low-quality reads yielded high-quality filtered reads (59.90%). The reads with a minimum PHRED quality score of <30 were filtered out. Fragment counting was performed in terms of counted fragments, unique fragments, non-specific fragments, and total fragments. The counted fragments were analyzed for further types as exon region, exon-exon region, total exon region, intron region, and total gene region. The total number of reads were reported as 2,99,47,616 for PG1 (Control sample of susceptible genotypes Bahar at 0 h), 3,51,58,120 for PG2 (Control sample of resistant genotypes KPL-44 at 0 h), 5,39,62,602 for PG3 (Treated sample of susceptible genotypes Bahar at 72 h), 2,96,73,844 for PG4 (Treated sample of resistant genotypes KPL-44 at 72 h), 4,41,66,980 for PG5 (Treated sample of susceptible genotypes Bahar at 96 h), and 3,68,03,128 for PG6 (Treated sample of resistant genotypes KPL-44 at 72 h), respectively as shown in Table 1. The input for reference genome of *C.cajan*V1.0 contains 31,841 genes and 38,885 transcripts with 12 assembled chromosomes and unplaced scaffolds. To analyze the transcript abundance assigned to a particular gene and, hence, the expression value transcript per gene, data were plotted out for all the transcriptomes. Most of the genes in all the transcriptomes were assigned to transcripts between 1 and 5 with a maximum abundance of two or three for a particular gene. To assess the contribution of transcripts toward coding region, exon per transcript value was determined and a nearly similar pattern for all the transcriptomes was observed, where maximum numbers of parent transcripts were annotating for 1–6 exons. The assembled transcripts were analyzed for length and it was found that most of the assembled transcripts were between 1,000 and 3,000 bp length and maximum transcripts were of ~2,000 bp length. After mapping to the reference genome 17589366, 26168786, 37450756, 20067116, 21302356, and 19730134 reads were mapped, respectively for PG1, PG2, PG3, PG4, PG5, and PG6. Maximum unmapped reads were observed for PG3 and minimum unmapped reads were reported for PG5. For Bahar, maximum read counts were observed at 72 h of infection followed by 96 h and 0 h of infection. The mapped reads constituted 58.73, 69.40, and 48.23% of total reads at 0, 72, and 96 hai. Similarly for KPL-44, maximum read counts were found at 92 h followed by 0 and 72 h of infection (Table 1). Further, maximum unique fragment count was obtained for PG2 at 72.7%, and least was for PG5. Similarly, total gene content for mapped reads was maximum in case of PG2 (0.73) and minimum for PG5 (0.52) (Table 1, Supplementary Data). Total counted fragments were ~99% for all the transcriptomes. Non-specifically counted fragments were abundantly found in PG5 and the least in PG2. Least uncounted fragments were reported for PG5 and maximum uncounted reads were noted in PG4. The mapped exon counting was maximum in PG2 (37.24%) followed by PG1 (27.06%) and least in PG4 (20.94%). Exon-exon content was highest in PG3 (10.68%) and lowest in PG5 (9.22%). Total exons were recorded at a maximum in PG2 (53.05%) and minimum in PG4 (27.18%). Therefore, intron content was reported least in PG2 and vice-versa. Moreover, the distribution of paired distance in all the transcriptomes was maximum in the range of 160–189 bp, except for PG5 where it was 160–264 bp (Figure 1).

**Table 1:** Statistic summary of the transcriptome data output with their NCBI submission Ids.

**S.No**	Sample Identity^*^	**Genotypes**	**Sample type**	**Read type**	**Read count**	**Reads mapped in pairs**	**Reads mapped in broken pairs**	**NCBI Project ID**
1	PG1	Bahar	Control (C, 0 h)	150 × 2 pair end	2,99,47,616	1,75,89,366	1,23,01,369	PRJNA688719
2	PG2	KPL-44	Control (C, 0 h)	150 × 2 pair end	3,51,58,120	2,61,68,786	89,39,641	PRJNA688719
3	PG3	Bahar	Treated (72 h, AI)	150 × 2 pair end	5,39,62,602	3,74,50,756	1,64,04,283	PRJNA688719
4	PG4	KPL-44	Treated (72 h, AI)	150 × 2 pair end	2,96,73,844	2,00,67,116	95,16,450	PRJNA688719
5	PG5	Bahar,	Treated (96 h, AI)	150 × 2 pair end	4,41,66,980	2,13,02,356	2,28,24,888	PRJNA688719
6	PG6	KPL-44	Treated (96 h, AI)	150 × 2 pair end	3,68,03,128	1,97,30,134	1,70,18,365	PRJNA688719

* *PG1: control sample of susceptible genotypes Bahar at 0 h; PG2: control sample of resistant genotypes KPL-44 at 0 h; PG3: Treated sample of susceptible genotypes Bahar at 72 h; PG4: Treated sample of resistant genotypes KPL-44 at 72 h; PG5: Treated sample of susceptible genotypes Bahar at 96 h; PG6: Treated sample of resistant genotypes KPL-44 at 72 h*.

Kal's Z statistical test was performed during comparison of the two cultivars at a different time of infection and in various combinations. The results were analyzed in terms of experiment fold change values, *P*-values, Bonferroni correction value, FDR, *p*-value correction, exon length, unique gene reads, total gene reads, unique exon reads, unique exon-exon reads, total exon-exon reads, unique intron reads, total intron reads, RPKM, chromosome start, and end region. Different comparisons were carried out among Bahar (PG1) vs. KPL-44 (PG2), Bahar (PG3) vs. KPL-44 (PG4) at 72 hai, Bahar (PG5) vs. KPL-44 (PG6), at 96 h AI, and within same genotype at different time intervals AI, for example between 0 and 72 h AI (Bahar, PG1 vs. PG3 and KPL-44, PG2 vs. PG4), 0 and 96 h AI (Bahar, PG1 vs. PG5 and KPL-44, PG2 vs. PG6), or 72 and 96 h AI (Bahar, PG3 vs. PG5 and KPL-44, PG4 vs. PG6). The comparison analysis determines the up-regulated and down-regulated genes differentially expressed between various transcriptomes in terms of gene and protein hits, gene ontology relationship, enrichment analysis, and pathway description using reference genomes as well as other organisms.

Combined, 2,720 gene locus were unique amongst PG1 vs. PG2, 860 were unique amongst PG1 vs. PG3, 1,193 gene locus were found on comparing PG1 vs. PG5, 1,770 in PG2 vs. PG6, and 52 were common to all (PG1 vs. PG2| PG1 vs. PG3| PG1 vs. PG5| PG2 vs. PG4| PG2 vs. PG6) in the up regulated transcripts. Similarly, in the down regulated gene locus, 1,862 were unique amongst PG1 vs. PG2, 501 were unique for PG1 vs. PG3, 509 for PG1 vs. PG5, 2,149 for PG2 vs. PG4, 1,139 for PG2 vs. PG6, and 23 were common to all (PG1 vs. PG2| PG1 vs. PG3| PG1 vs. PG5| PG2 vs. PG4| PG2 vs. PG6).

**Figure 2:**
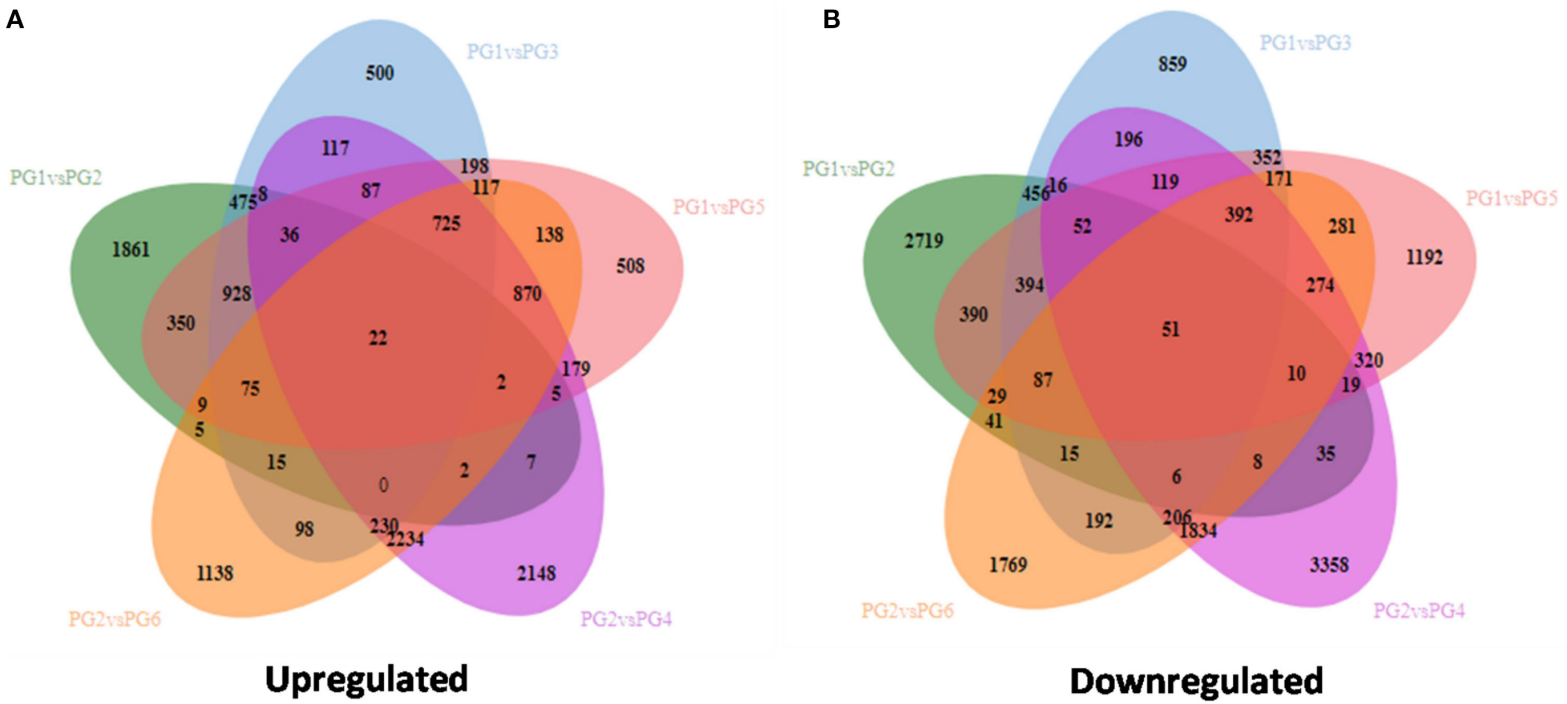
Venn diagram for comparison of various transcriptome for differentially expressed genes **(A)** Up regulated genes **(B)** Down regulated genes.

During different points of infection, specifically for the susceptible genotype, 1,452 proteins were up regulated and 1,431 proteins were down regulated in PG1 vs. PG3 and 1.672 proteins were up regulated and 1.627 proteins were down regulated in PG1 vs. PG5. Similarly, for the resistant genotype, 2,243 proteins were up regulated and 2,430 proteins were down regulated in PG2 vs. PG4 and 1,940 proteins were up regulated and 2,080 proteins were down regulated in PG2 vs. PG6. Moreover, on comparing the two genotypes at different infection times, 1,762 gene hits were obtained in PG1 vs. PG2, 1,365 gene hits were obtained in PG3 vs. PG4, and 9,460 gene hits were obtained in PG5 vs. PG6 (Figure 2).

Taking into consideration the differential gene expression data of pigeon/pea wilt susceptible/tolerant control and infected transcriptomes, genes such as defensin-like protein, disease resistance proteins viz. At4g27190, RGA1, RGA2, RGA3, RGA4, RPP13, RPM1, RPP8, and RPM1, major pollen allergen, mildew resistant protein, MLO-like protein, MLP-like protein, pro-hevein, stress-induced protein SAM, wound-induced protein WIN, S-norcoclaurine synthase, TMV resistant protein, and so on may be potential candidate genes as the proposed leads for future molecular based analysis of this crop. These probable putative genes and prevalent associated pathways in response to pathogen infection could be explored in signaling molecules in host-pathogen interaction and different metabolic pathways with roles in inherent resistance mechanisms. Through further research, we could functionally validate and regulate their expression either through gene editing or over expression to develop a resistant cultivar.

## Data Availability Statement

The raw reads for all the Illumina sequenced transcriptome used for the analysis have been deposited to NCBI with the BioProject ID PRJNA688719.

## Author Contributions

KS and NS conceived the idea and provided critical input to the concept. ST and MH contributed to analysis, statistical analysis, data interpretation, and wrote the manuscript. SP, PG, SA, and RM conducted the experiments. AB, KK, AK, PS, and MA contributed resources, manuscript editing, and interpretation. All the authors read the manuscript and agreed on publication.

## Conflict of Interest

The authors declare that the research was conducted in the absence of any commercial or financial relationships that could be construed as a potential conflict of interest.
