# Supplementary material for: Comparative Time Series RNA-seq Analysis of Pigeonpea Root Tissues in Response to Fusarium udum Infection
Source: Front Fungal Biol. 2021 May 17;2:664953. doi: 10.3389/ffunb.2021.664953 (PMC10512240; doi:10.3389/ffunb.2021.664953)

Generated by: root
Date: Thu Aug 17 19:45:10 IST 2017

Table of contents

1. Selected input sequences 3
    1.1 Sequence reads 3
    1.2 Reference Sequences 3
2. References 3
    2.1 Transcripts per gene 3
    2.2 Exons per transcript 4
    2.3 Length of transcripts 5
3. Mapping statistics 5
    3.1 Paired reads 5
    3.2 Fragment counting 6
    3.3 Counted fragments by type (total) 6
    3.4 Match specificity (paired) 7
    3.5 Paired distance 8

1. Selected input sequences

1.1 Sequence reads

Name	Number of reads	Longest read	paired
PG1	29,947,616	150	yes

For 'paired' data, there are two 'reads' in a pair.

1.2 Reference Sequences

References	Length	Genes	Transcripts
2,957	535,755,142	31,841	38,885

2. References

Input contained 31,841 genes and 38,885 transcripts.

2.1 Transcripts per gene

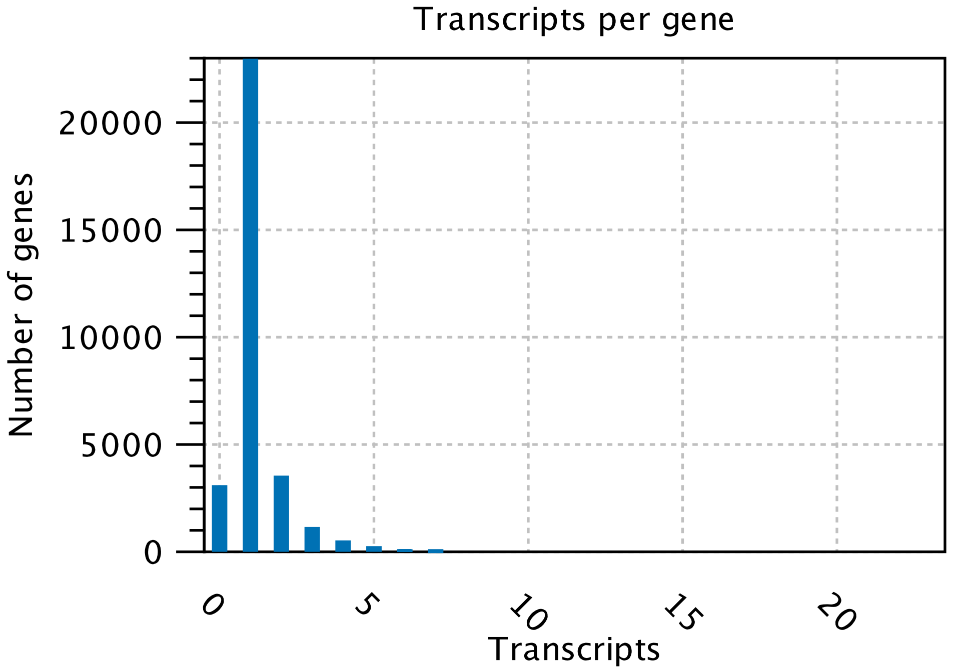

2.2 Exons per transcript

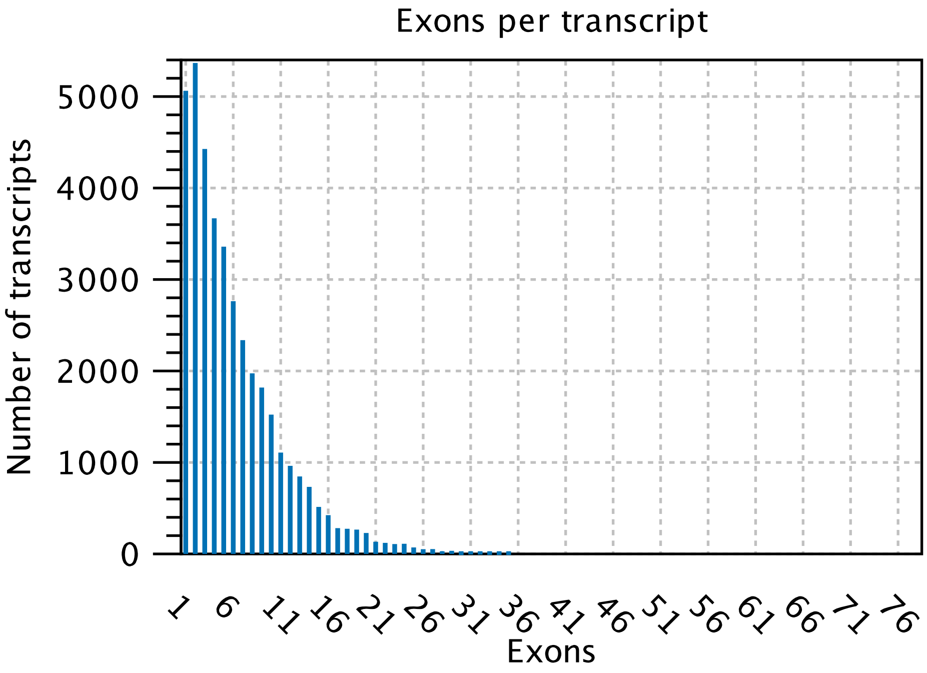

2.3 Length of transcripts

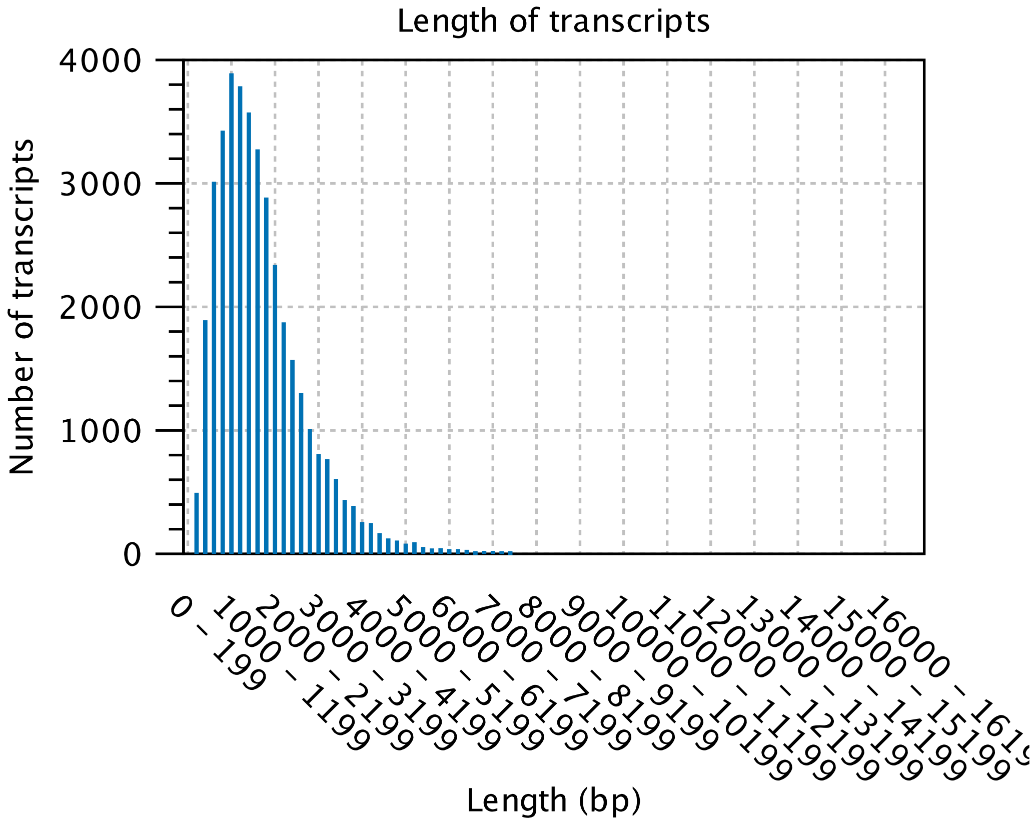

3. Mapping statistics

3.1 Paired reads

	Number of sequences	%
Reads mapped in pairs	17,589,366	58.73
Reads mapped in broken pairs	12,301,369	41.08
Reads not mapped	56,881	0.19
Total	29,947,616	100.00

For 'paired' data, there are two 'reads' in a pair.

3.2 Fragment counting

	Single	% of Total	Paired	% of Total
Counted fragments	0	0.00	29,890,735	100.00
- unique fragments	0	0.00	19,996,543	100.00
- non-specifically	0	0.00	9,894,192	100.00
Uncounted fragments	0	0.00	56,881	100.00
Total fragments	0	0.00	29,947,616	100.00

Total	% of Total Fragments
29,890,735	99.81
19,996,543	66.77
9,894,192	33.04
56,881	0.19
29,947,616	100.00

'Include broken pairs' counting scheme: A pair is counted as two, a single read as one

3.3 Counted fragments by type (total)

	Uniquely mapped	Fraction	Non-specifically mapped	Fraction
Exon	7,459,486	0.92	629,441	0.08
Exon-exon	2,555,256	0.91	247,490	0.09
Total exon	10,014,742	0.92	876,931	0.08
Intron	9,981,801	0.53	9,017,261	0.47
Total gene	19,996,543	0.67	9,894,192	0.33

Mapped	% of total mapped
8,088,927	27.06
2,802,746	9.38
10,891,673	36.44
18,999,062	63.56
29,890,735	100.00

'Include broken pairs' counting scheme: A pair is counted as two, a single read as one

3.4 Match specificity (paired)

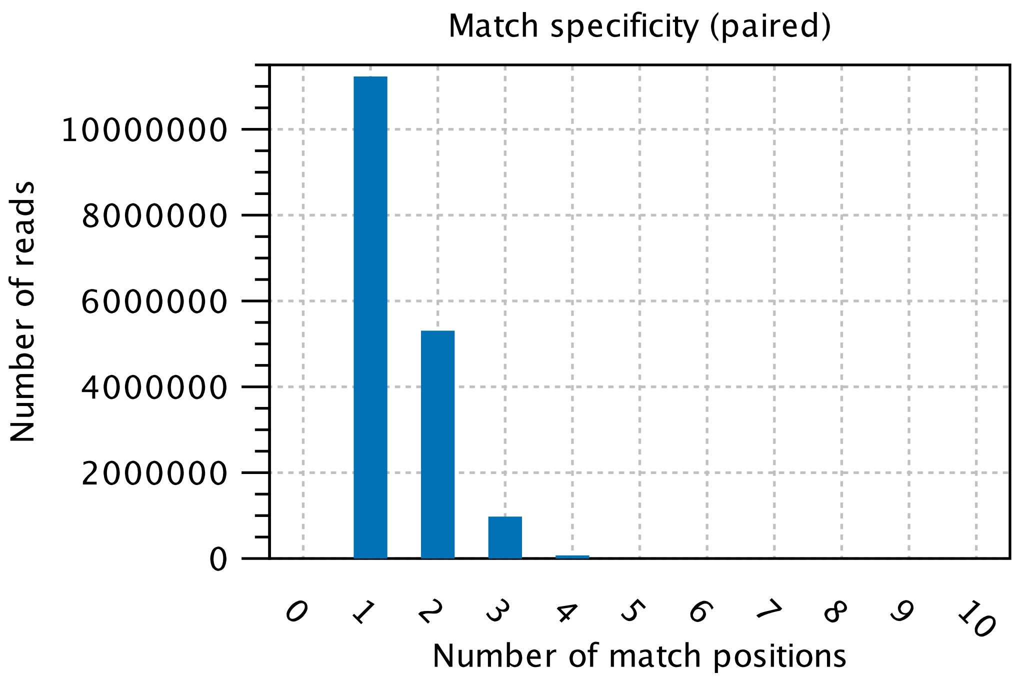

3.5 Paired distance

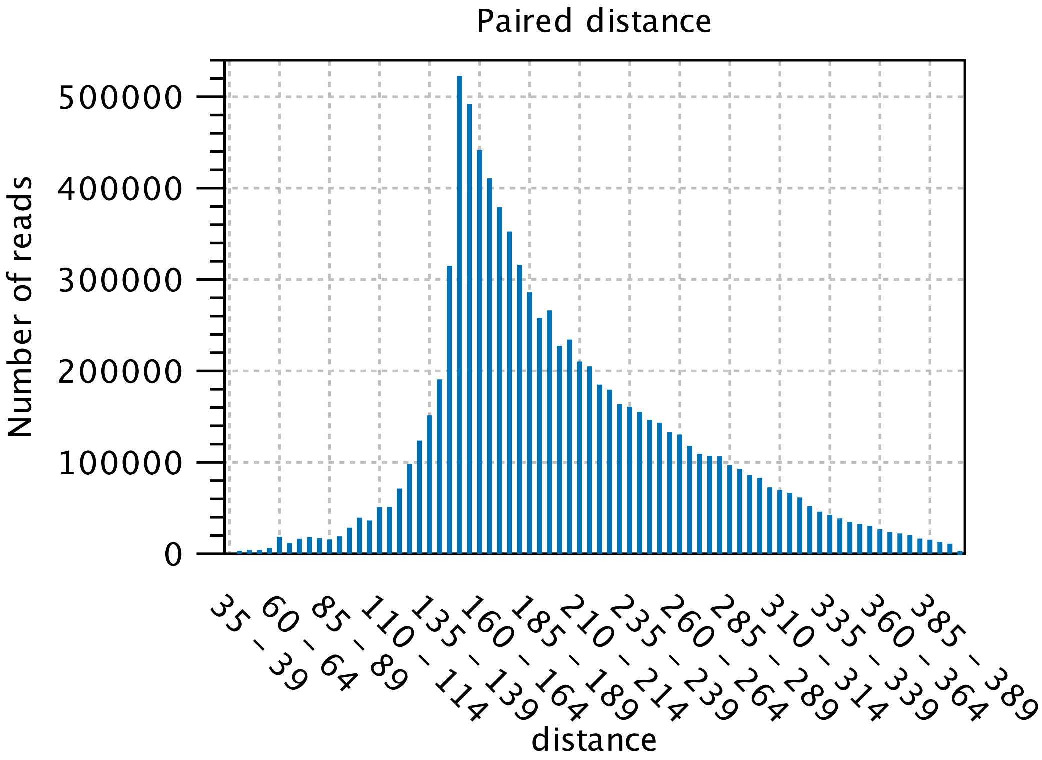

Supplement: Supplementary file 1 [file Data_Sheet_1.PDF]
