# Supplementary material for: Comparative Time Series RNA-seq Analysis of Pigeonpea Root Tissues in Response to Fusarium udum Infection
Source: Front Fungal Biol. 2021 May 17;2:664953. doi: 10.3389/ffunb.2021.664953 (PMC10512240; doi:10.3389/ffunb.2021.664953)
Supplement: Supplementary file 2 [file Data_Sheet_2.doc]

| **NGS Experiment Overview:** | |
| --- | --- |
| Organism | : Plant |
| Sequence length | : 101 nt |
| Adapter | : Illumina Truseq RNA seq adapter |
| Protocol | : Illumina TruSeq |
| NGS Platform used | : Illumina HiSeq2500 |
| Tool used for QC | : NGS QC Tool kit, MS Excel |
| Library layout | : Paired End |


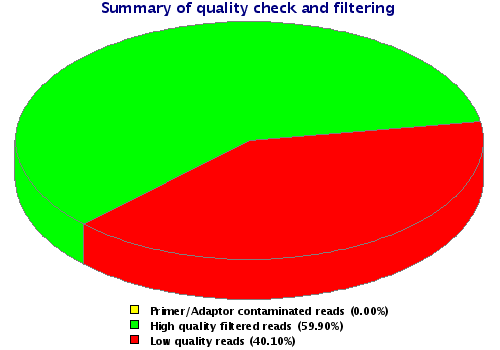


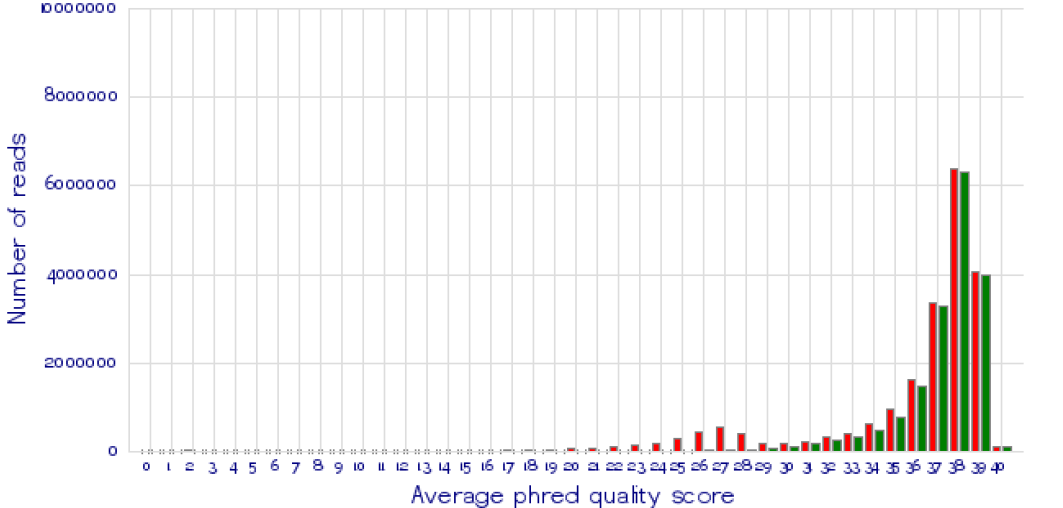
Fig 2. Pie chart shows the summary of QC depicting percentage of high quality, low quality and contaminated reads.

Fig 3. Quality distribution of number of reads for different average PHRED quality score.
